# A Cell Internalizing Antibody Targeting Capsid Protein (p24) Inhibits the Replication of HIV-1 in T Cells Lines and PBMCs: A Proof of Concept Study

**DOI:** 10.1371/journal.pone.0145986

**Published:** 2016-01-07

**Authors:** Syed A. Ali, Sin-Yeang Teow, Tasyriq Che Omar, Alan Soo-Beng Khoo, Tan Soo Choon, Narazah Mohd Yusoff

**Affiliations:** 1 Oncological and Radiological Sciences, Advanced Medical and Dental Institute, Universiti Sains Malaysia, 13200, Kepala Batas, Pulau Pinang, Malaysia; 2 Institute for Medical Research, Jalan Pahang, 50588, Kuala Lumpur, Malaysia; 3 Institute for Research in Molecular Medicine (INFORMM), Universiti Sains Malaysia, 11800, Pulau Pinang, Malaysia; 4 Regenerative Medicine, Advanced Medical and Dental Institute, Universiti Sains Malaysia, 13200, Kepala Batas, Pulau Pinang, Malaysia; Institute of Medicinal Biotechnology, Chinese Academy of Medical Sciences, CHINA

## Abstract

There remains a need for newer therapeutic approaches to combat HIV/AIDS. Viral capsid protein p24 plays important roles in HIV pathogenesis. Peptides and small molecule inhibitors targeting p24 have shown to inhibit virus replication in treated cell. High specificity and biological stability of monoclonal antibodies (mAbs) make them an attractive contender for in vivo treatments. However, mAbs do not enter into cells, thus are restricted to target surface molecules. This also makes targeting intracellular HIV-1 p24 a challenge. A mAb specific to p24 that can internalize into the HIV-infected cells is hypothesized to inhibit the virus replication. We selected a mAb that has previously shown to inhibit p24 polymerization in an in vitro assay and chemically conjugated it with cell penetrating peptides (CPP) to generate cell internalizing anti-p24 mAbs. Out of 8 CPPs tested, κFGF-MTS -conjugated mAbs internalized T cells most efficiently. At nontoxic concentration, the κFGF-MTS-anti-p24-mAbs reduced the HIV-1 replication up to 73 and 49% in T-lymphocyte and PBMCs respectively. Marked inhibition of HIV-1 replication in relevant cells by κFGF-MTS-anti-p24-mAbs represents a viable strategy to target HIV proteins present inside the cells.

## Introduction

Human immunodeficiency virus type-1 (HIV-1) infections remain a major global threat. Continuous efforts in anti-HIV-1 drugs and vaccine development have failed to eliminate or prevent HIV/AIDS [1]. Albeit the combination antiviral therapy known as highly active antiretroviral therapy (HAART) is able to suppress the disease progression to a certain extent, AIDS morbidity and mortality remain high due to the emergence of drug-resistant HIV-1 escape mutants [2]. Therefore, there is a pressing need to explore new approaches for developing effective treatment modalities against HIV/AIDS [1, 2].

Capsid plays important roles in both early and late stages of HIV-1 replication cycle [3]. After viral entry into host cells, the intact cone-shaped capsid is released into cell cytosol, and disassembles in a coordinated fashion to allow reverse transcription and genome integration for an effective establishment of HIV infection [3]. During the virus particle assembly, capsid domains interact with Gag polyprotein that undergoes proteolytic cleavage, and transform the immature particles to mature virions. Upon formation of mature virions, ~1,500 p24 monomers assemble into a lattice of p24 hexamers and pentamers that packages the viral RNA genome and other proteins [4].

HIV-1 capsid protein p24 is a relatively underexplored target for the development of HIV-1 inhibitors that has attracted considerable attention in recent years. Pivotal findings that host restriction factors such as Trim5α target incoming capsid core emphasize the potential of p24 as an anti-HIV target [5, 6, 7]. Tang and coworkers were the first to demonstrate the potential of targeting p24 using small molecule inhibitor called CAP-1 [8]. This work was followed by several publications reporting the inhibition of p24 by small inhibitor molecules [9, 10, 11, 12, 13, 14, 15, 16, 17] and peptides [18, 19, 20] both in vitro and in infected cells. These reports show that p24 can be a potential target for development of HIV/AIDS therapy.

Monoclonal antibodies (mAbs) are highly specific and biologically stable molecules, making them an attractive candidate for in vivo treatments. However, antibodies do not readily permeate cell membranes, thus their therapeutic actions are largely limited to target surface antigens [21]. This also makes targeting HIV-1 p24, which is an intracellular protein, a challenge. Cellular import of proteins has been accomplished by molecularly engineering proteins with short membrane transport facilitating peptides called cell penetrating peptides (CPPs) [22]. Zhao and co-workers chemically conjugated the IgG molecule with a CPP called κFGF-MTS and showed that the resulting κFGF-MTS-mAb conjugates (they coined a term ‘TransmAbs’) efficiently internalized into living 3T3 fibroblast cells [23]. In a subsequent report they showed that anti-active caspase-3 antibody when conjugated with κFGF-MTS peptide could internalize into Jurkat T cells and inhibit actinomycin D -induced apoptosis and prevent the cleavage of alpha II spectrin [24]. In another report, a κFGF-MTS -conjugated anti-Pyk2 mAb has shown to be internalized into SF-767 human glioblastoma cells where it efficiently inhibited glioma cell migration [25]. Similarly, a κFGF-MTS -conjugated anti-ricin A chain antibody internalized into RAW 264.7 murine macrophage cells and significantly inhibited ricin -induced toxicity [26]. These reports demonstrated that normal IgG can be engineered into cell internalizing antibodies using a simple conjugation technique and the resulting TransmAbs could internalize into a variety of cells and target intracellular proteins.

We have previously reported that a hybridoma -derived anti-p24 mAb was able to inhibit the p24 polymerization in an in vitro assay [27]. We hypothesized that the same antibody when conjugated with CPPs would internalize into T cells and inhibit HIV replication. To test our hypothesis, we covalently attached the anti-p24 mAb with various CPPs, and assessed their internalization into T cells. Among eight CPP-anti-p24 mAbs tested, κFGF-MTS -conjugated anti-p24 mAb exhibited maximum internalization into Jurkat and H9 T cells and was subsequently used to assess the HIV-1 inhibition in the same cells and PBMCs. Our data show that κFGF-MTS-anti-p24 mAbs can internalize into T cells and markedly inhibit HIV-1 replication. These results indicate that CPP-conjugated mAbs can be used as vehicles to target HIV-related proteins inside the cells.

## Materials and Methods

### Cells lines and antibodies

Jurkat T Clone E6-1 (#177), H9 T (#87), MAGI-CCR5 (#3522), and HIV-1 p24 Hybridoma 183-H12-5C (#1513) cells were obtained through the NIH AIDS Reagent Program, Division of AIDS, NIAID, NIH. Primary peripheral blood mononuclear cells (#PCS-800-011), HEK293T (#CRL-3216), HIV-1 p24 Hybridoma 31-90-25 (#HB-9725), and AE-1 Hybridoma (#HB-72) cells were purchased from American Type Culture Collection (ATCC). Jurkat and H9 T cell lines were maintained in RPMI 1640 supplemented with 2 mM L-glutamine and 10% FBS at 37°C in a 5% CO_2_ incubator. Antibody -secreting hybridoma cell lines were propagated in DMEM supplemented with 10% FBS at 37°C in a 5% CO_2_ incubator. Primary peripheral blood mononuclear cells (PBMCs) were maintained in RPMI 1640 supplemented with 10% FBS and 20 U/mL IL2 (#11697; through the NIH AIDS Reagent Program, Division of AIDS, NIAID, NIH) at 37°C in a 5% CO_2_ incubator. The mAbs were purified from cell culture supernatants by proteinG affinity chromatography using HiTrap ProteinG HP columns pre-packed with ProteinG Sepharose (GE, #17-0404-01) on an FPLC system (AKTA purifier 900, GE). The purified antibodies were then analyzed by SDS-PAGE, dialyzed against phosphate buffered saline (PBS) using Slide-A-Lyzer Dialysis Cassette, 7K MWCO (Thermo Scientific, #66710), and stored at -80°C in small aliquots.

### Synthesis of antibody-peptide conjugates

Cell penetrating peptides (**Table 1**) were synthesized at >90% purity by BioBasic Canada. Lyophilized peptides were dissolved in sterile water to final concentration of 10 mg/mL and stored at -80°C in 100 μL aliquots. Antibodies (anti-p24 clone 31-90-25 and anti-AchE clone AE-1) were conjugated with CPPs essentially as described by Zhao and co-workers [23, 24]. Briefly, antibodies were concentrated to 2 mg/mL by Pierce Concentrator 20 K MWCO (Thermo Scientific, #89886A) and dialyzed against 1 L PBS (pH 6.0) for 1 h at 22°C. The antibodies were then oxidized by adding sodium periodate (BioBasic Canada, #SB0875) to final concentration of 200 mM and incubating on a rocking platform for 30 min at 4°C in dark. Glycerol, to final concentration of 30 mM, was added to stop the reaction and oxidized antibodies dialyzed against 1 L PBS (pH 6.0) for 1 h at 4°C. Peptides were added to 100 X molar excess over antibody and reaction incubated for 1 h at 37°C. Unconjugated peptides were removed by dialyzing the antibodies against 1 L PBS (pH 7.2) using Slide-A-Lyzer Dialysis Cassette, 7 K MWCO. Resulting CPP-mAbs were fluorometrically quantitated using Qubit Fluorimeter (Life technologies), analyzed by SDS-PAGE to determine their banding patterns, and subjected to ELISA to assess their binding to cognate antigens.

**Table 1:** Cell-penetrating peptides (CPPs) used for cell internalizing antibody engineering.

Peptide	Sequence	Size kDa)	pH	pI	Charge
BMV Gag	KMTRAQRRAAARRNRWTAR	2.36	Basic	13.3	8
FHV Coat	RRRRNRTRRNRRRVR	2.16	Basic	13.5	11
HIV-1D-Tat	GRKKRRQRRRPPQ	1.72	Basic	13.2	8
HIV-R9-Tat	GRRRRRRRRRPPQ	1.80	Basic	13.4	9
HTLV-II Rex	TRRQRTRRARRNR	1.78	Basic	13.4	8
κFGF-MTS	KAAVALLPAVLLALLP	1.57	Basic	9.69	1
Penetratin	RQIKIWFQNRRMKWKK	2.25	Basic	12.8	7
sC3d-P16	KNRWEDPGKQLYNVEA	1.95	Neutral	6.62	0

Physicochemical properties of CPPs were analyzed by online tool ‘Peptide Property Calculator’. Source: https://www.genscript.com/ssl-bin/site2/peptide_calculation.cgi

### Evaluation of antibody internalization by ELISA

Antibody internalization was assessed by antibody capture ELISA as previously reported by Zhao and co-workers [23] with few modifications. Jurkat or H9 T cells were plated into 24-well culture plate at a density of 1x10^5^ cells/well in 0.5 mL complete culture medium. Antibodies (1 μg/well) were added and cells incubated at 37°C in a 5% CO_2_ incubator for 3, 6, 12, and 18 h. In some experiments cells were incubated with graded concentration (0.1, 0.5, 1, 5, 10, and 20 μg) of antibodies and incubated for 18 h. At each time point, cells were transferred to microfuge tubes and centrifuged at 400 x g for 5 min at 4°C. Same number of cells were re-suspended in PBS (pH 7.4)/Trypsin (1 mg/mL) and incubated for 15 min [28] at 37°C followed by 2X washing with cold PBS (pH 7.4). This step was carried out to remove non-internalized surface-bound antibodies. Cells were then re-suspended in 100 μL cold PBS/1X Halt Protease Inhibitor Cocktail (Life Technologies, #78430) and homogenized by Pellet Pestle Motor (Kontes) for 30 sec. Cell homogenate was centrifuged at 12,000 x g for 30 min at 4°C and clear supernatant was stored at -20°C until use.

The wells of the ELISA microtiter plate (Nunc, #442404) were coated with HIV-1 p24 antigen (1 μg/mL), diluted in 20 mM carbonate buffer pH 9.6 and each well received 100 μL. The p24 antigen was allowed to adsorb for overnight at 22°C after which the wells were washed 2X with wash buffer (PBS pH 7.4, 0.05% Tween-20). The wells of the plate were blocked with 200 μL/well of blocking buffer (PBS pH 7.4, 5% skimmed milk) for 2 h at 22°C after which the wells were washed 4X with wash buffer. Clarified cell homogenates (diluted 1:10 with PBS) were added to the wells (100 μL/well). After incubation for 1 h at 37°C and 4X washing, 0.5 μg/mL anti-mouse IgG-HRP (KPL, #474–1806) was added to the wells (100 μL/well) and plate incubated for 1 h at 22°C. Wells were washed 4X and TMB substrate solution (KPL, #52-00-01) was added to each well (100 μL/well) and plate incubated for 30 min at 22°C. The color development was stopped by adding 1M sulfuric acid (100 μL/well) and its optical density (OD) was measured at a wave length of 450 nm with reference wavelength of 670 nm.

### Determination of Cytotoxicity

The cytotoxicity of antibodies was assessed against the tested cell lines by using Trypan blue exclusion assay. Cells were plated into 24-well plate at a density of 1 x 10^5^ cells per well and co-cultured with different concentrations of antibodies at 37°C in a 5% CO_2_ incubator for 18 h. Next, the cells were diluted 1:1 with 0.4% Trypan blue solution (Gibco, #15250) and counted by hemocytometer and the viability determined.

### Live cell confocal microscopy

The κFGF-MTS-conjugated anti-p24 mAbs were labelled with Fluorescein using Pierce NHS-Fluorescein Antibody Labeling Kit (Thermo Scientific #53029) following the suppliers' instructions. Cells were co-cultured with Fluorescein -labelled κFGF-MTS-anti-p24 mAb at 37°C for 18 h. The cells were then treated with trypsin and washed with PBS to remove non-internalized surface-bound antibodies. Cells were resuspended in Hank's balanced salt solution (HBSS) containing 2% FBS and 20 mM HEPES. A small aliquot (10–20 μL) of cell suspension was placed on a microslide and a 18 x 18 mm glass coverslip was mounted on the sample. Confocal images were obtained using a Nikon Ti-Eclipse inverted motorized microscope equipped with Nikon A1Rsi spectral detector confocal system running NIS-C Elements software (Version 4.20). The cells were observed using Nikon Plan Apo VC 60x Oil immersion objective. The cells were approximately 12–15 μm in size and Z-slices were obtained every 1 micron. For excitation of FITC the (488 nm laser line) of an (Argon-ion laser) was used. Sequential scanning mode was used to avoid bleed-through between the channels; laser and photomultiplier settings were kept constant within each experiment. Image optimization of contrast, gain and brightness was performed to the same extent within each experiment and intensities of the respective panels are therefore comparable.

### Preparation of HIV-1 (NL4.3) virus stocks

To prepare HIV-1 NL4.3 virus stocks, endotoxin -free pNL4.3 (#114; through the NIH AIDS Reagent Program, Division of AIDS, NIAID, NIH) was used to transfect HEK293T cells using XtremeGene HP transfection reagent (Roche, #06366236001) following supplied protocol. At two days post-transfection, culture supernatants were harvested and centrifuged at 500 x g for 10 min at 4°C. Cell -free supernatants were then passed through pre-washed (with PBS) 0.4 μm syringe filter (Sartorius Stedim, #16533) to remove residual cellular contaminants. Filtered supernatants were concentrated by ultracentrifugation in OptimaTM L-100K (Beckman Coulter) using an SW41 rotor at 100,000 x g for 90 min at 4°C. The pellet was washed 1X with sterile PBS and then incubated in the presence of 0.5 mL serum-free DMEM for overnight at 4°C. Next day, the tube contents were mixed by gentle pipetting and concentrated virus stock was stored at -80°C in small aliquots. MAGI (an HIV-1 LTR-β-gal reporter cell line) assay was performed for quantitative virus concentration and transduction unit (TU) titration.

### MAGI-CCR5 assay

MAGI-CCR5 cells were plated into 96-well culture plate (Corning, #3628) at a density of 1x10^4^ cells/well in 0.1 mL complete medium (DMEM supplemented with 10% fetal bovine serum, 300 μg/ml L-glutamine, 0.2 mg/ml G418; 0.1 mg/ml hygromycin B; and 1 μg/ml puromycin). Next day, cells were infected by removing the medium and adding virus in 300 μL of complete DMEM (without G418/hygromycin) with 20 μg/mL DEAE-dextran (BioBasic Canada, #DB0147). After 2 h incubation (at 37°C in a 5% CO_2_ incubator), 0.2 mL DMEM/10%FBS was added and cells incubated for 2 days at 37°C in a 5% CO_2_ incubator. Culture medium was removed and 0.2 mL fixing solution (1% formaldehyde, 0.8% gluteraldehyde in PBS) was added to each well. Cells were fixed for 5 min at 21°C and washed 2X with PBS (pH7.4). Cells were then stained by adding 80 μL staining solution (4 mM potassium ferrocyanide (Sigma-Aldrich, #P9387), 4 mM potassium ferricyanide (Sigma-Aldrich, #244023), 2 mM MgCl_2_, and 0.4 mg/mL X-gal (BioBasic Canada, #BB0083) in PBS) to each well and incubating at 37°C for 50 minutes in a non-CO_2_ incubator. The staining solution was removed and wells were washed 3X with PBS (pH 7.4). Blue foci were counted using inverted microscope and the transduction units were expressed as the number of blue cells per μL virus stock.

### Infection assays in T cell lines

CD4 -expressing Jurkat or H9 T cells were dispensed into sterile 5 mL round bottom cell culture tube (BD Falcon, #352054) at a density of 2x10^5^ cells in 1 mL complete medium. Antibody was added and cultures incubated at 37°C in a 5% CO_2_ incubator for 18 h. Cells were then centrifuged at 400 x g for 5 min and washed 3X with cold PBS (pH7.4) to remove non-internalized surface-bound antibodies. Cells were then re-suspended in 0.1 mL complete medium and infected with HIV-1 NL4.3 virus at 0.1 multiplicity of infection (MOI) for 2 h at 37°C in a 5% CO_2_ incubator; mixing cells every 30 min. Next, 0.9 mL of complete medium was added and cultures incubated at 37°C in a 5% CO_2_ incubator for 48 h. Culture supernatant was harvested by centrifuging cells at 400 x g for 5min. Virus particles present in spent medium were pelleted through a 20% (w/v in PBS) sucrose cushion (to remove soluble proteins including antibodies) by ultracentrifugation [29] in Optima Max (Beckman Coulter) using a MLS-50 rotor at 100,000 x g for 90 min, at 4°C. Concentrated virions were re-suspended in original volume in serum-free DMEM and used for MAGI assay as described above.

In some experiments cells were exposed to antibodies continuously. To do that, Jurkat or H9 T cells were dispensed into culture tubes at 2x10^5^ cells, treated with antibodies for 18 h, washed 3 X with cold PBS and infected with HIV-1 (NL4.3) for 2 h. Complete culture medium with antibody was then added to the culture tubes and cells incubated for another 48 h. Culture supernatants were prepared as described above and used for MAGI assay.

### Pull-down assay and immunoblot analysis

Jurkat or H9 T cells were incubated with 10 μg of κFGF-MTS-anti-p24 mAb for 18 h. Cells were washed to remove non-internalized surface-bound antibodies and either infected with HIV-1 NL4.3 at 0.1 MOI or mock infected as described above. Infected cells were incubated for 48 h after which cells were washed 3X with cold PBS. Cells (5 x 10^6^) were lysed and centrifuged at 10,000 x g for 10 min. Clarified cell lysate was incubated with 25 μL ProteinG magnetic beads (NEB, # S1430S) at 4°C for 60 min. Immunoprecipitates were collected by applying magnetic field for 30 sec. Cell lysate was removed and magnetic beads were washed 3X with supplied immunoprecipitation buffer. To separate immunoprecipitates from magnetic beads, the samples were incubated at 70°C for 5 min and placed in a magnetic field. Bead-free supernatant was collected and used for SDS-PAGE and Western blot analyses. For immunoblot analysis, blots were probed with anti-p24 mAb (clone 183-H12-5C). The blots were blocked, washed, and incubated with HRP-conjugated anti-mouse IgG.

### Infection assays in PBMCs

PBMCs were spun down at 500 x g for 10 min at 22°C and re-suspended in activation medium (RPMI 1640, 10% FBS, 5 μg/mL PHA-M (Sigma-Aldrich, #L8902)) at a concentration of 1x10^6^ cells/mL. Cells were incubated for three days at 37°C in a 5% CO_2_ incubator, centrifuged at 500 x g for 10 min and washed once with RPMI 1640. Cells were then re-suspended in complete growth medium (RPMI 1640, 10% FBS, 20 U/mL IL2) and added to 5 mL culture tubes at a concentration of 2x10^5^ cells in 1 mL medium. Antibody was added and cells incubated at 37°C in a 5% CO_2_ incubator for 18 h. Cells were then centrifuged at 400 x g for 5 min and washed 3 X with cold PBS (pH7.4) to remove unbound antibodies. Cells were re-suspended in 0.1 mL complete medium and infected with HIV-1 NL4.3 virus at 0.1 MOI for 2 h at 37°C in a 5% CO_2_ incubator; mixing cells every 30 min_._ Next, 0.9 mL of complete medium (with antibody) was added and cells incubated at 37°C in a 5% CO_2_ incubator. Cells were fed every 2 days by removing half of the medium. This medium was used to prepare antibody-free virions for MAGI assay as described above. Cells were replenished with fresh medium containing antibodies while cells density was maintained at 1–2 x 10^5^ cells per tube.

In some experiments, cells were infected with HIV-1 prior to exposing to antibodies. To do that, cells were infected with HIV-1 for 2 h, followed by the addition of complete medium (without antibodies). Cells were incubated for 24 h at 37°C in a 5% CO_2_ incubator, centrifuged at 400 x g for 10 min, and washed 2 X with cold PBS. Cells were re-suspended in fresh culture medium containing 10 μg/mL antibody and incubated at 37°C in a 5% CO_2_ incubator. Cells were fed every 2 days by removing half of the medium. This medium was used to prepare antibody-free virions for MAGI assay as described above. Cells were replenished with fresh medium containing antibodies while cells density was maintained at 1–2 x 10^5^ cells per tube.

### Statistical Analysis

All experiments were performed independently in duplicate on at-least three occasions. All values are expressed as the mean ± standard deviations. Statistical comparisons were performed using a Student’s t-test by IBM SPSS Statistics Version 21 software. A *p*-value of less than 0.05 was considered statistically significant.

## Results

### Affinity cross-linking of CPPs to antibodies

Eight CPPs **(Table 1)** were synthesized and cross-linked via carbohydrate moieties to two mAbs i.e. anti-p24 (clone 31-90-25) and anti-AchE (clone AE-1). Because glycosylation sites in mAbs are predominantly found on the Fc region of the antibody, they can be safely modified without significantly affecting the antigen-binding capacity [21, 23]. The resulting CPP-anti-p24 and CPP-anti-AchE mAbs were analyzed by SDS-PAGE. No differences in the banding of heavy and light chains between CPP-mAbs and native mAbs were observed (data not shown). The affinity of the CPP-mAbs towards their cognate antigens was also analyzed by ELISA and compared with native mAbs. No differences were observed between the ODs obtained for CPP-mAbs and native mAbs (data not shown).

### Internalization of CPP-mAbs into cells

**Fig 1:**
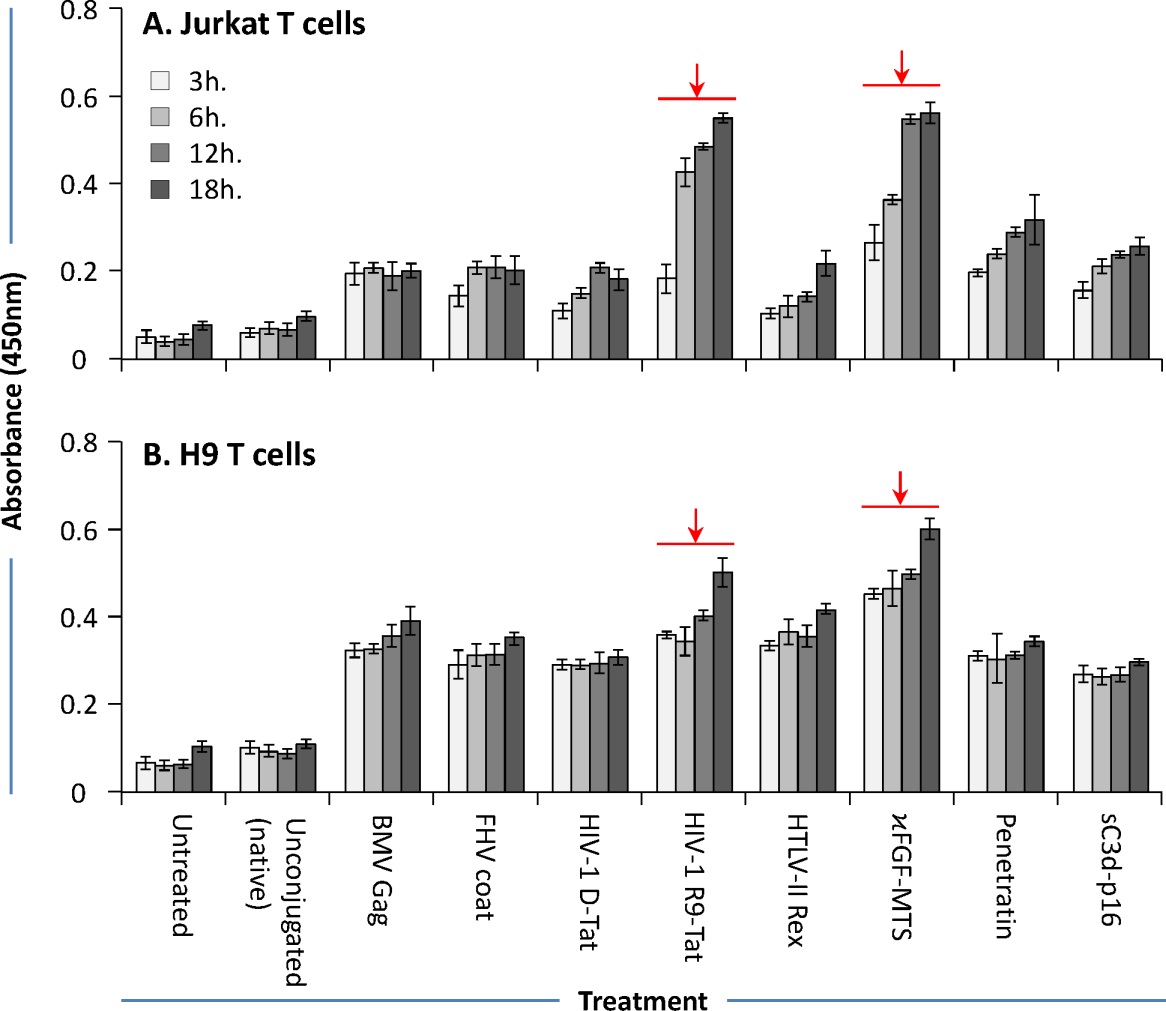
Internalization of CPP-conjugated anti-p24 mAbs into Jurkat and H9 T cells. Cells were co-cultured with CPP-anti-p24 mAbs at 37°C for 18 h. Cells were then treated with trypsin and washed with cold PBS before lysing. The cell lysates were centrifuged and clarified cell supernatants were used to determine CPP-anti-p24 mAb internalization by antibody capture ELISA as described by Zhou and co-workers [23]. The results are representative of three independent experiments each done in duplicate. **(A)** CPP-anti-p24 mAb internalization into Jurkat T cells. **(B)** CPP-anti-p24 mAb internalization into H9 T cells.

The ability of CPP-mAbs to internalize into cells was studied using co-culture experiments followed by antibody capture ELISA as described by Zhao and co-workers [23, 24]. Jurkat or H9 T cell lines were cultured in the presence of either native (anti-p24 mAb) or CPP-anti-p24 mAbs for various times. To assess the antibody internalization, cells were treated with trypsin and washed to remove non-internalized surface-bound antibodies. Cells were then lysed, and diluted cell lysates were analyzed by indirect p24 ELISA designed to capture anti-p24 mAbs. Significantly more antibodies were detected in lysates from cells that were co-cultured with CPP-anti-p24 mAbs compared to those that were incubated together with native anti-p24 mAbs (**Fig 1**). Out of eight CPP-anti-p24 mAbs, κFGF-MTS and HIV-R9-Tat -conjugated anti-p24 mAb showed maximum internalization in both tested cell lines (**Fig 1A and 1B**). Internalization of CPP-anti-p24 mAbs increased with time during the 18 h incubation, particularly in Jurkat T cells. Significantly less antibody was detected in lysates from cells that were co-cultured with unconjugated control anti-p24 mAbs. These data support the role of CPPs, particularly κFGF-MTS and HIV-1 R9-Tat in efficiently transporting the anti-p24 mAbs into the cells (shown by high ODs). Due to most efficient internalization, κFGF-MTS-anti-p24 mAb was used in subsequent experiments.

### Concentration -dependent antibody internalization

**Fig 2:**
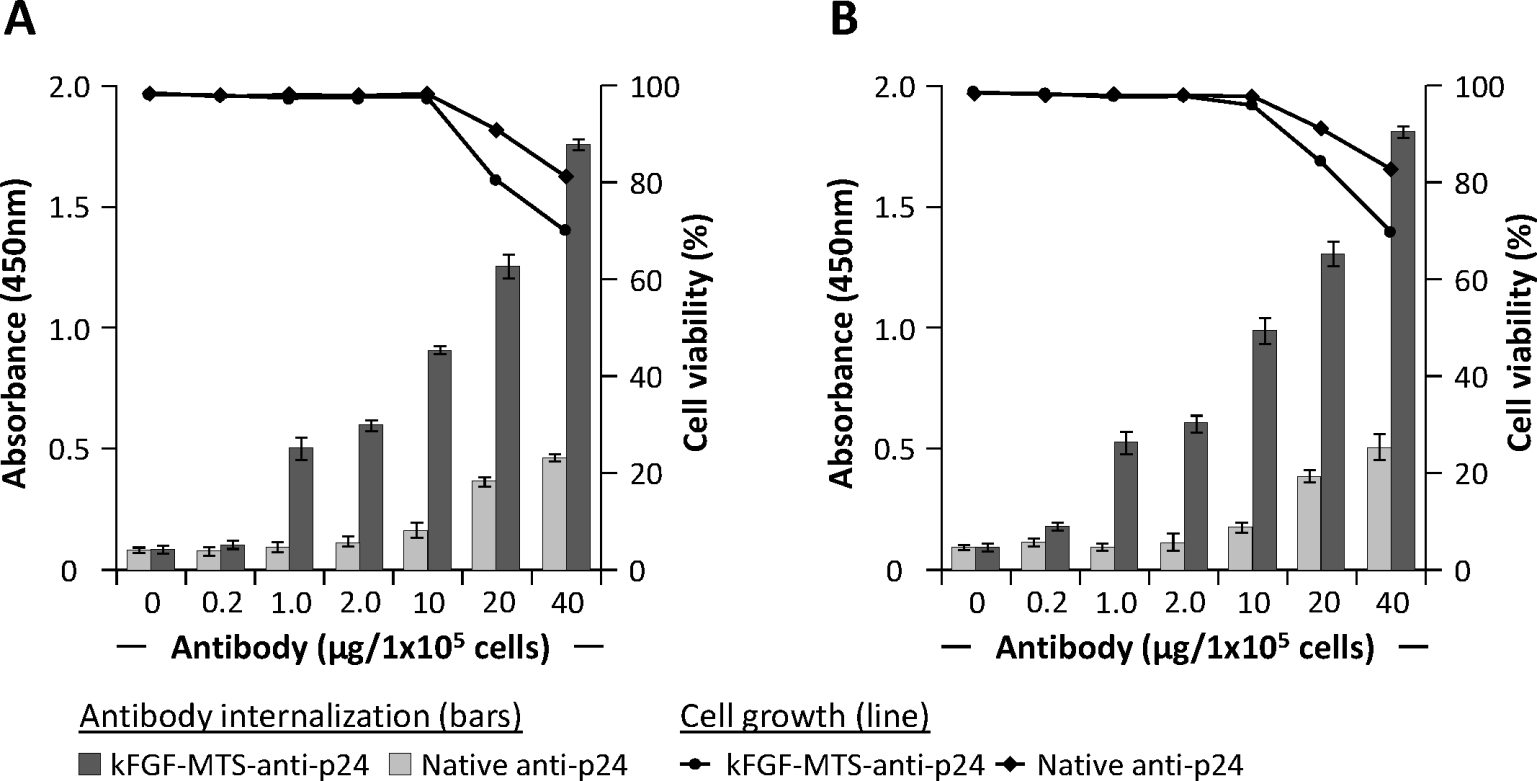
Concentration -dependent internalization of κFGF-MTS-anti-p24 mAb into T cells. Cells were incubated with indicated concentrations of κFGF-MTS-anti-p24 mAbs or native anti-p24 mAb at 37°C for 18 h. Cells were treated with Trypsin and washed to remove non-internalized antibodies. Washed cells were homogenized and clarified cell lysates were subjected to antibody capture ELISA for the determination of antibody internalization. Bars show antibody internalization into cells whereas lines show cell viability. The results are representative of three independent experiments each done in duplicate. **(A)** Antibody capture ELISA and cell viability on Jurkat T cell lysates. **(B)** Antibody capture ELISA and cell viability on H9 T cells.

In above experiment we incubated 1x10^5^ cells with 1 μg native or CPP-anti-p24 mAbs and demonstrated internalization of CPP -conjugated antibodies into the cells. In this experiment we incubated Jurkat and H9 T cells with varying concentrations of native or κFGF-MTS-anti-p24 mAbs for 18 h and determined antibody internalization. Treated cells were also tested for toxicity. The antibody capture ELISA detected significantly more antibodies in lysates from cells that were incubated with higher concentrations of CPP-anti-p24 mAb (**Fig 2A and 2B)**. Compared to Jurkat, lysates from H9 T cells gave higher OD signals in antibody capture ELISA suggesting that κFGF-MTS-anti-p24 mAbs internalized into H9 T cells relatively more efficiently. Antibody capture ELISA also detected antibodies in lysates from cells that were incubated with higher concentrations of native antibodies. Some internalization of native IgGs after prolonged incubation with cells has also been reported by Zhou and co-workers [24]. To test whether κFGF-MTS-anti-p24 mAbs were toxic, cell viability was determined by using Trypan blue cell exclusion assay. Incubation with up to 10 μg κFGF-MTS -conjugated or unconjugated antibodies had no effect on viability of cells. However, cells treated with 20 and 40 μg of κFGF-MTS -conjugated or unconjugated antibodies showed reduced viability (**Fig 2A and 2B**).

**Fig 3:**
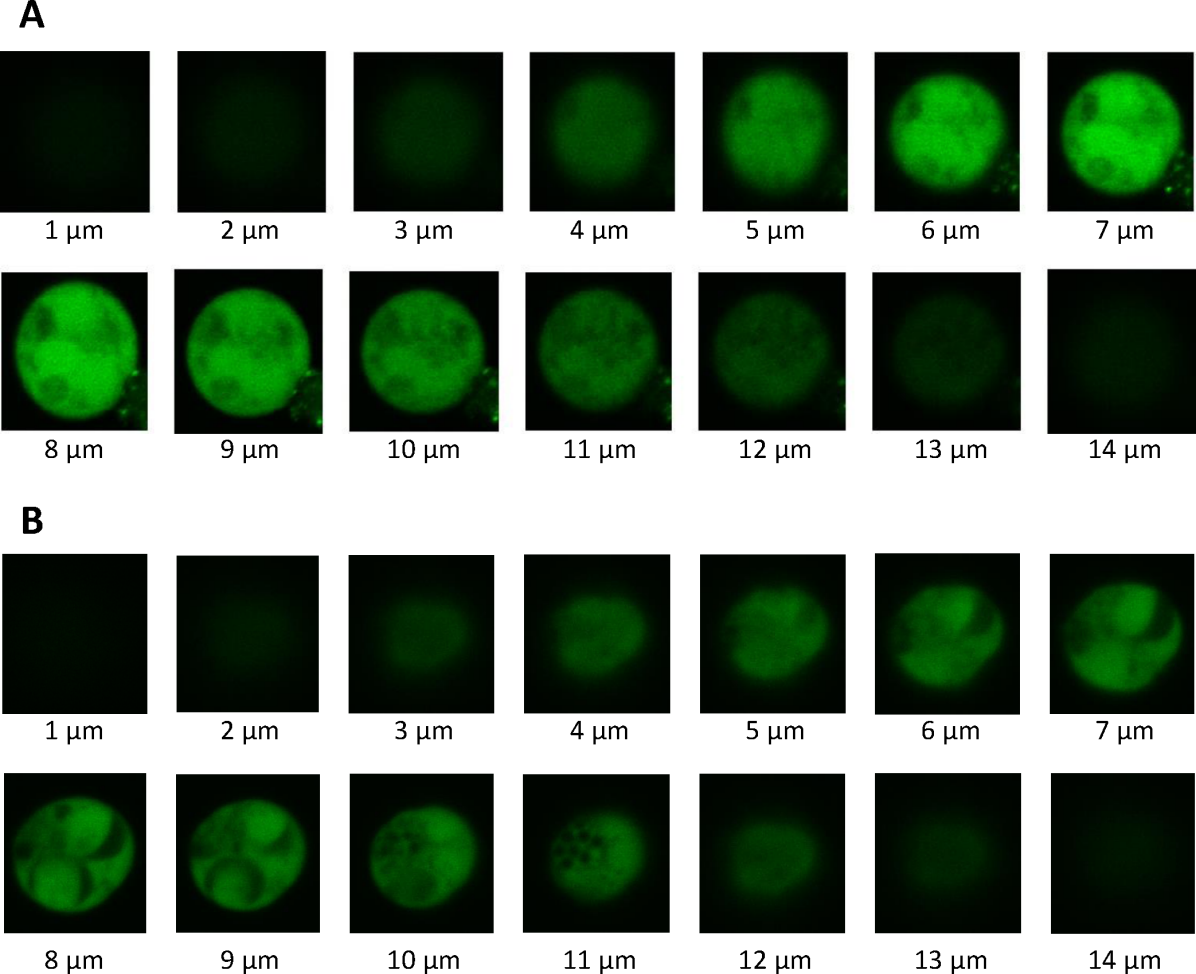
Laser confocal microscopy of cells treated with κFGF-MTS-anti-p24 mAb. Cells were co-incubated with 10 μg Fluorescein -labelled κFGF-MTS-anti-p24 mAb at 37°C for 18 h. Cells were treated with trypsin and washed to remove non-internalized antibodies. Confocal images were obtained using a Nikon Ti-Eclipse inverted motorized microscope equipped with Nikon A1Rsi spectral detector confocal system running NIS-C Elements software (Version 4.20). A series of Z-stack images shows the presence of internalized antibodies into the cytoplasm of the cells. **(A)** Fluorescein -labelled κFGF-MTS-anti-p24 mAb internalization into Jurkat T cells. **(B)** Fluorescein -labelled κFGF-MTS-anti-p24 mAb internalization into H9 T cell.

To confirm internalization of κFGF-MTS-anti-p24 mAb into cells, Jurkat and H9 T cells were incubated with Fluorescein -labelled κFGF-MTS-anti-p24 mAb for 18 h. Cells were treated with trypsin to remove non-internalized surface bound antibodies, and observed using a confocal microscope. Z stack images were collected at 1 μm intervals and range from 1 to 14 μm. The κFGF-MTS-anti-p24 mAbs appeared to localize inside the cells (**Fig 3A and 3B**).

### κFGF-MTS-anti-p24 mAb inhibits the virus replication in T cells

**Fig 4:**
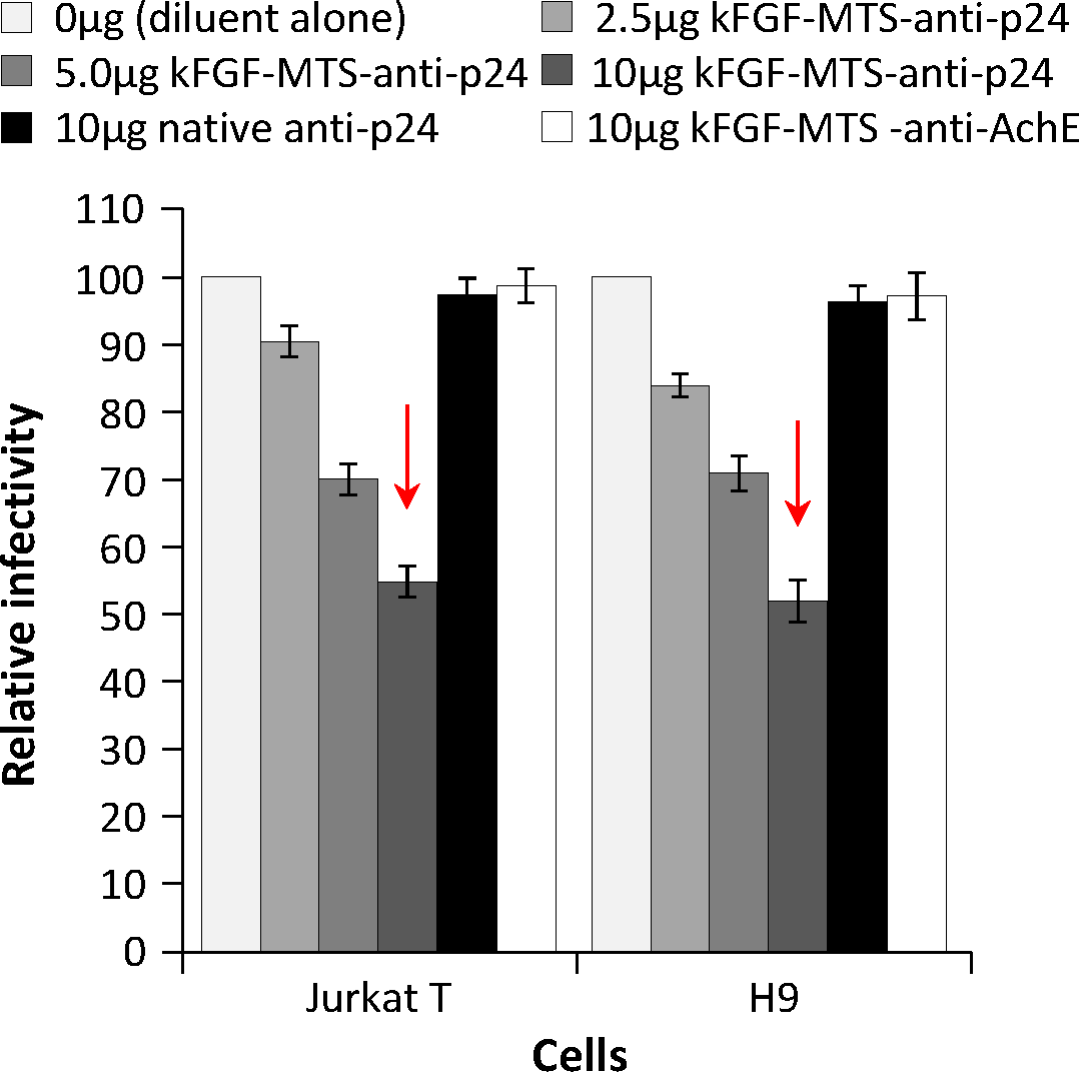
κFGF-MTS-anti-p24 mAb inhibits the virus replication in treated cells. Cells were incubated with indicated concentrations of κFGF-MTS-anti-p24 mAb, native anti-p24 mAb, and unrelated MTS-anti-AchE mAb at 37°C for 18 h. Cells were washed to remove unbound antibody and infected with HIV-1 NL4.3 at MOI 0.1 for 2 h. Complete culture medium was then added and cells were cultured for 48 h. Culture supernatant was harvested and used to infect MAGI-CCR5 cells. Two days after infection, MAGI-CCR5 cells were fixed, stained and blue foci counted. The results are representative of three independent experiments each done in duplicate.

To test if internalized κFGF-MTS-anti-p24 mAbs would inhibit virus replication, we incubated Jurkat and H9 T cells with graded concentration (0, 2.5, 5, 10 μg/mL) of κFGF-MTS-anti-p24 mAbs for 18 h, washed the cells to remove non-internalized surface-bound antibodies, and challenged the antibody -treated cells with laboratory-adapted X4-tropic HIV-1 NL4.3 at 0.1 MOI for 6 h. In control experiments, cells were incubated with native anti-p24 or unrelated κFGF-MTS-anti-AchE mAbs prior to challenge. To test the virus production by treated cells, culture supernatants were harvested 48 h post-challenge and used to infect MAGI-CCR5 cells, an HIV-1 LTR-β-gal reporter cell line. To ensure that antibodies present in the culture supernatant were not interfering with MAGI assay, we pelleted down the virions through a 20% sucrose cushion that removes soluble proteins from the virus preparations [29]. The culture supernatants produced from cells that were treated with various concentrations of κFGF-MTS-anti-p24 mAb showed a dose -dependent reduction in virus replication as determined by MAGI cell assay. Virus replication was inhibited by ~50% in cells that were treated with 10 μg/mL κFGF-MTS-anti-p24 mAb (**Fig 4**). In contrast, no reduction in virus replication was observed for cells that were treated with native anti-p24 mAb or unrelated κFGF-MTS-anti-AchE mAbs. These data demonstrate that only HIV-1 p24-specific and κFGF-MTS-conjugated cell internalizing antibody was capable of attenuating the virus replication in Jurkat and H9 cells.

**Fig 5:**
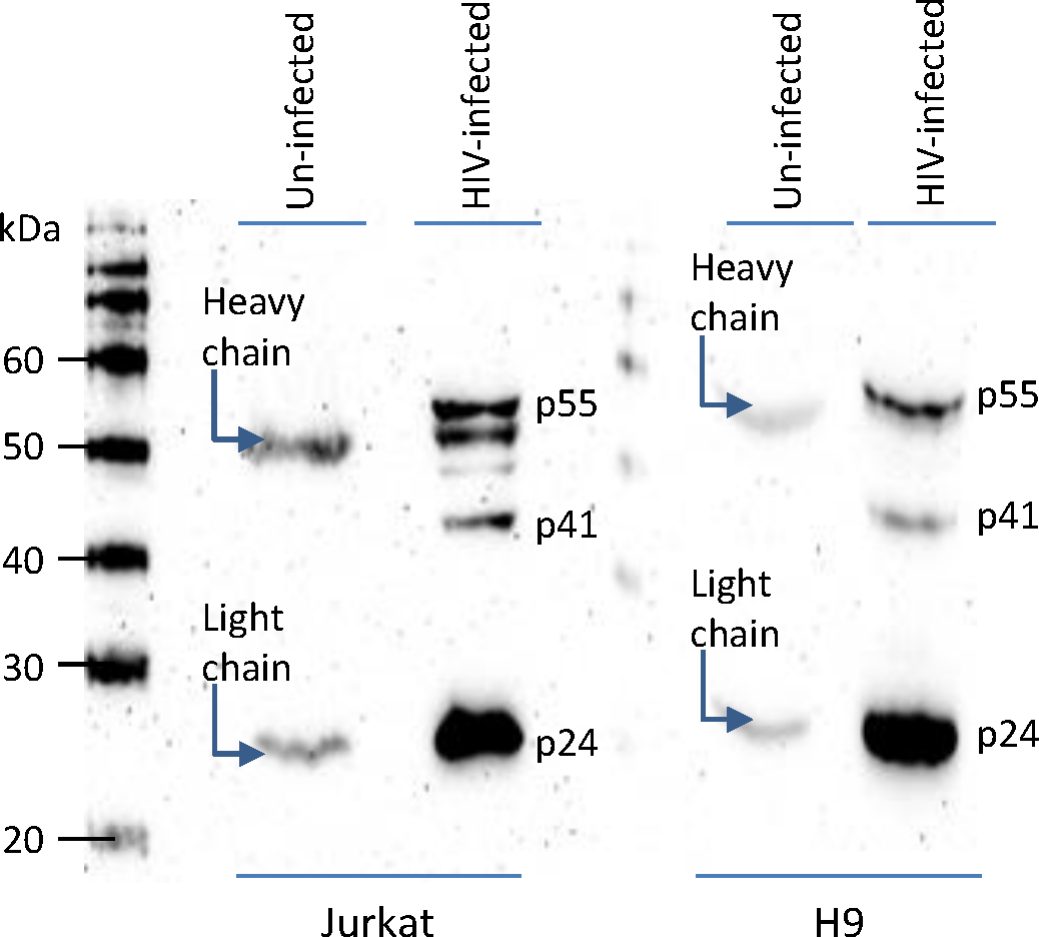
Association of κFGF-MTS-anti-p24 mAb with HIV Gag. Cells were incubated with indicated concentrations of κFGF-MTS-anti-p24 mAb at 37°C for 18 h. Cells were washed to remove unbound antibody and either infected with HIV-1 NL4.3 at MOI 0.1 for 2 h or mock infected (control). Complete culture medium was then added and cells were cultured for 48 h. Cells were washed, lysed, and clarified cell lysates were incubated with ProteinG magnetic beads. Magnetically captured immunoprecipitates were analyzed by Western blotting.

To ascertain the association of κFGF-MTS-anti-p24 mAb with HIV Gag in the infected cells, we performed a pull-down assay. We co-incubated Jurkat or H9 T cells with κFGF-MTS-anti-p24 mAbs and then infected the cells with HIV-1 as described above. Cells were lysed and internalized κFGF-MTS-anti-p24 mAbs were captured using ProteinG magnetic beads. When probed with an anti-p24 mAb (clone 183-H12-5C), lanes loaded with immunoprecipitates from HIV- infected cells were positive for p55, p41, and p24 bands (**Fig 5**). This confirmed that internalized κFGF-MTS-anti-p24 mAb interacted with HIV Gag protein.

### Continuous exposure of cells to κFGF-MTS-anti-p24 mAb enhances the reduction of virus replication

**Fig 6:**
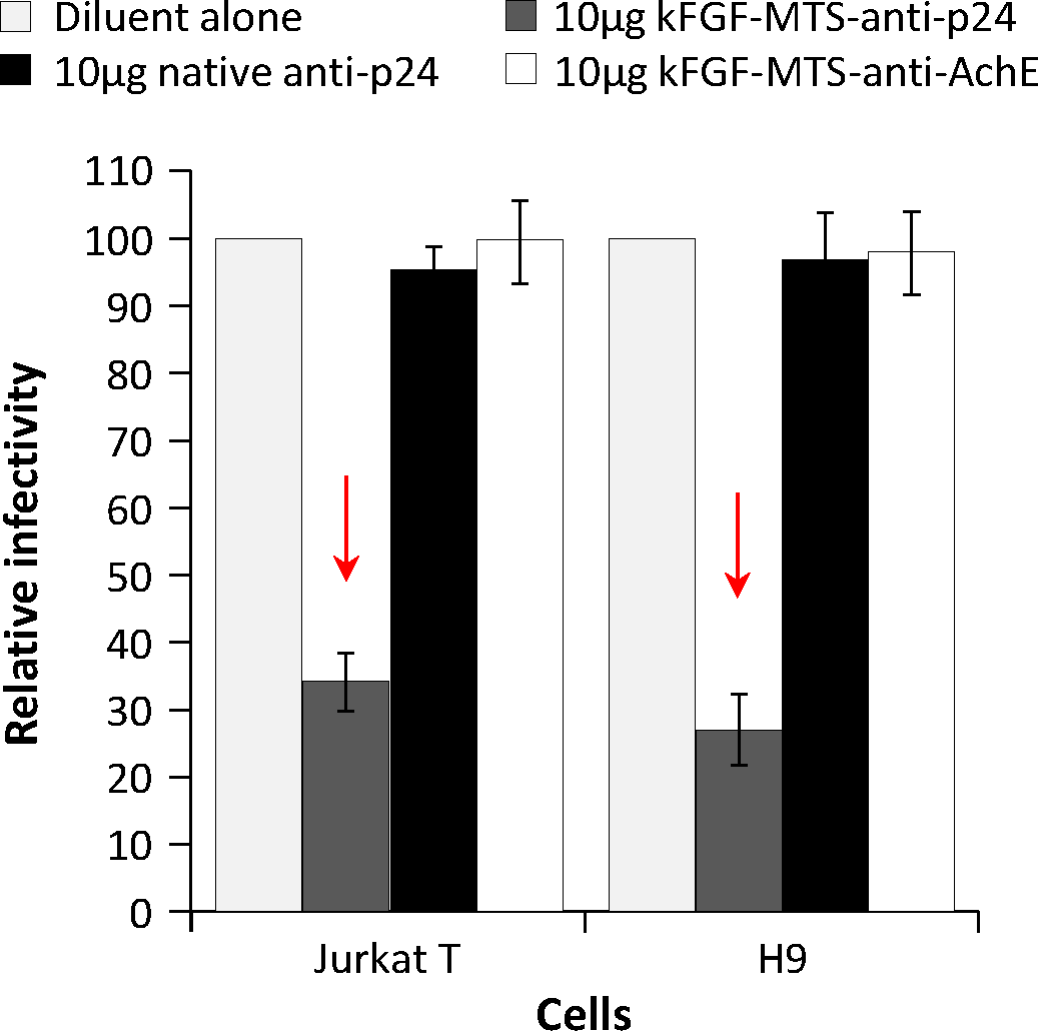
Continuous exposure to antibodies enhances antiviral activity of κFGF-MTS-anti-p24 mAbs. Cells were incubated with 10 μg/mL of κFGF-MTS-anti-p24 mAb, native anti-p24 mAb, and unrelated κFGF-MTS-anti-AchE mAb at 37°C for 18 h. Cells were then washed and infected with HIV-1 NL4.3 at MOI 0.1 for 2 h. Complete culture medium containing 10 μg/mL of antibodies was added and cells incubated for 48 h after which culture supernatants were collected and used to infect MAGI cells. Two days after infections, MAGI cells were fixed, stained and blue foci counted. The results are representative of three independent experiments each done in duplicate.

In this experiment we investigated whether continuous exposure of cells to antibody would enhance the reduction of virus replication. We incubated Jurkat and H9 T cells with10 μg/mL of κFGF-MTS-anti-p24 mAbs for 18 h, washed the cells, and challenged with HIV-1 NL4.3 at 0.1 MOI. In control experiments, cells were incubated with native anti-p24 mAb or an unrelated κFGF-MTS-anti-AchE mAbs at 10 μg/mL for 18h prior to challenge. Complete culture medium containing 10 μg/mL of antibodies (test or control) was added and cells incubated for 48 h after which culture supernatant was collected and used to infect MAGI-CCR5 cells. Compared to single 18 h exposure **(Fig 4)**, the continuous treatment of cells with κFGF-MTS-anti-p24 mAb resulted in relatively better inhibition of HIV-1 replication in Jurkat (66%) and H9 T (73%) cells **(Fig 6)**. Consistent with results of previous experiment, cell treatment with native anti-p24 mAb or unrelated κFGF-MTS-anti-AchE mAb did not reduce the virus replication appreciably. These data show that κFGF-MTS-anti-p24 mAb was able to continuously suppress the HIV-1 replication throughout the 3-day course.

### κFGF-MTS-anti-p24 mAb effectively reduces HIV-1 replication in primary PBMCs

**Fig 7:**
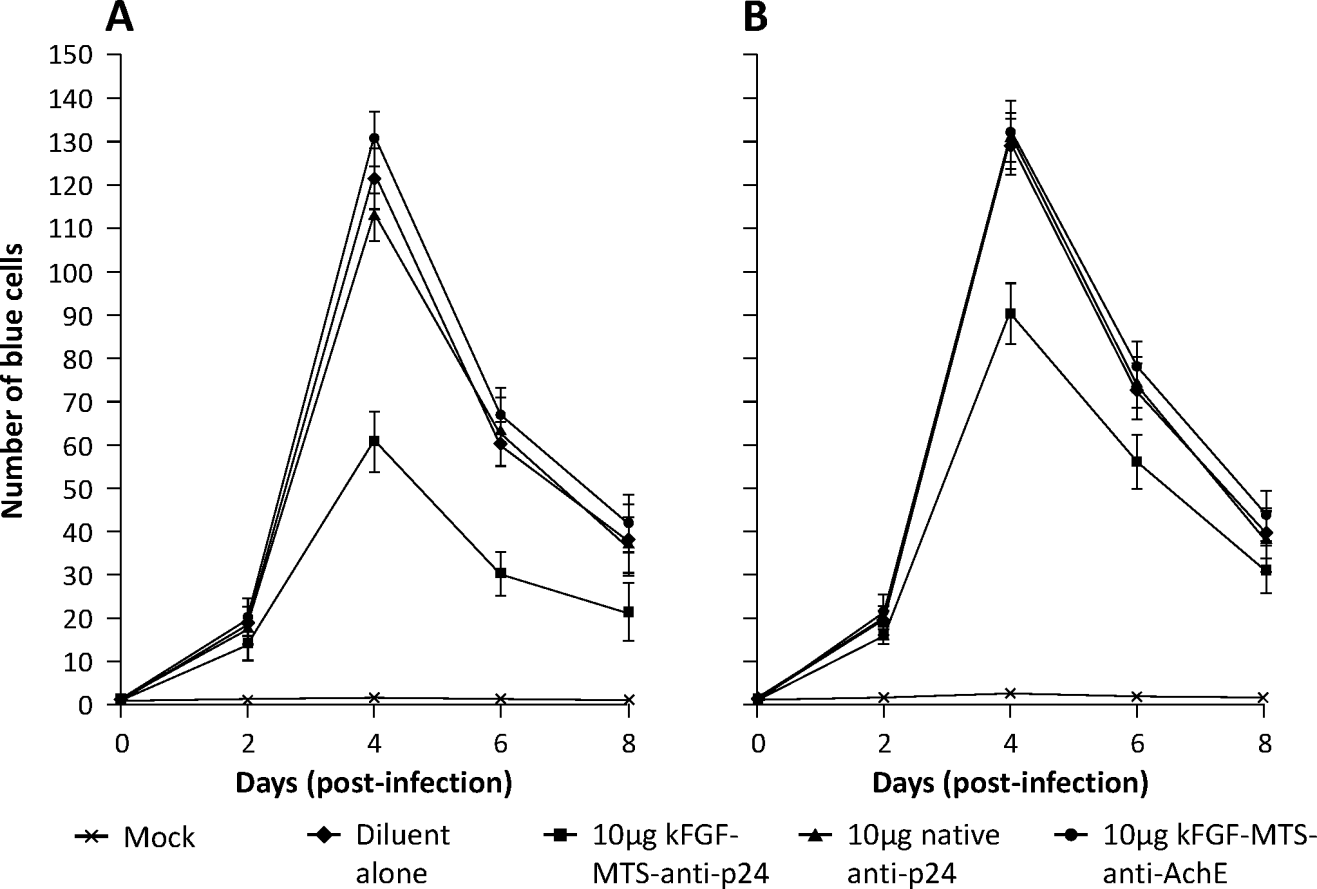
κFGF-MTS-anti-p24 mAb reduces HIV-1 infection of PBMCs. **(A)** PHA -activated PBMCs were incubated with 10 μg/mL of κFGF-MTS-anti-p24 mAbs, native anti-p24 mAb, or unrelated κFGF-MTS-anti-AchE mAbs at 37°C for 18 h. Cells were washed and challenged with HIV-1 NL4.3 at MOI 0.1 for 2 h. Cells were co-cultured with 10 μg/mL of antibodies for up to 8 days and fed with fresh medium with antibodies every 48 h. Spent medium was harvested for MAGI assay. At each harvest, the cells were counted and re-plated to normalize the viral production at per cell base. The virus replication was quantitatively determined by counting the blue foci and plotted. **(B)** Activated PBMCs were infected with HIV-1 for 2 h. After 24 h, cells were treated with antibodies for up to 8 days. Cells were replenished with fresh medium containing antibodies every 48 h whereas spent medium was harvested for MAGI assay. The results are representative of three independent experiments each done in duplicate.

To validate our findings in a more relevant setting, we co-cultured PHA -activated primary PBMCs with 10 μg/mL of κFGF-MTS-anti-p24 mAbs for 18 h, and challenged with HIV-1 NL4.3 at 0.1 MOI. In control experiments, PBMCs were treated with 10 μg/mL native anti-p24 mAb or an unrelated κFGF-MTS-anti-AchE mAbs for 18 h prior to challenge. Following challenge, cells were re-suspended in culture medium containing antibodies and incubated for 48 h. At each harvest, cells were counted and the same number of viable cells was plated and fed with fresh media containing 10 μg/mL antibodies (test or control) for up to 8 days. Harvested spent medium was used to infect MAGI-CCR5 cells. Infectivity kinetics demonstrated markedly reduced HIV-1 replication (up to 49%) in primary PBMCs that were treated with κFGF-MTS-anti-p24 mAbs compared to cells that were treated with native anti-p24 or un-related κFGF-MTS-anti-AchE mAbs (**Fig 7A**). Percentage of inhibition was maintained throughout eight days. However, overall inhibition of HIV-1 replication in PBMCs was relatively low compared to T-cell lines.

Until now we had tested the efficacy of MTS-anti-p24 mAbs against HIV-1 replication in cells that were exposed to κFGF-MTS-anti-24 mAbs prior to infection. Virus replication inhibition in these cells suggested that κFGF-MTS-anti-p24 mAb was targeting early stages of infection. We were interested to find out whether κFGF-MTS-anti-p24 mAb could also target late stages of infection. It has been shown that HIV-1 integration is a rapid process and integrated HIV DNA starts accumulating within 1 h post-infection [30]. We infected PBMCs with HIV-1 for 24 h, after which the infected cells were co-incubated with antibodies as described in **Materials and methods**. Infectivity kinetics showed approximately 30% reduced HIV-1 replication in primary PBMCs that were treated with κFGF-MTS-anti-p24 mAbs compared to those that were treated with native anti-p24 mAb or un-related κFGF-MTS-anti-AchE mAbs (**Fig 7B**). Inhibition of HIV replication in cells that were infected before antibody treatment was markedly low (30%) compared to cells that were incubated with MTS-anti-p24 mAbs prior to HIV-1 infection (49%). This suggests that MTS-anti-p24 mAb targets early stages of infection more efficiently compared to late stages.

## Discussion

We have previously shown that hybridoma -derived anti-p24 mAb was able to inhibit the capsid polymerization in an in vitro assay [27]. We hypothesized that same antibody, when conjugated with CPPs, would internalize into T cells, interfere with p24 polymerization, and subsequently inhibit HIV-1 replication. To test our hypothesis, we chemically conjugated abovementioned anti-p24 mAb with various CPPs, and assessed the internalization of resulting CPP-anti-p24 mAbs into Jurkat and H9 T cells by using antibody capture ELISA. CPP-anti-p24 mAbs internalized into both cell types in a time dependent manner and reached plateau in 18 h post-exposure. Out of eight CPP-anti-p24 mAbs, κFGF-MTS and HIV-1 R9-Tat -conjugated anti-p24 mAbs internalized into both cells types relatively more efficiently. In contrast, lysates from cells that were co-incubated with native (not conjugated with CPPs) mAbs did not show high OD absorbance in the antibody capture ELISA. These results also confirm the observations of Zhao and co-workers who have previously reported the internalization of a κFGF-MTS -conjugated anti-caspase-3 mAb into Jurkat T cells [24]. Zhao and co-workers have shown that internalization of antibodies into the cells could be monitored by using antibody capture ELISA [23, 24]. Washing cells twice with cold PBS appeared to be sufficient to remove non-internalized surface-bound antibodies and did not interfere with ELISA readings [23, 24]. In optimization experiments, we compared cold PBS washings with 5 and 15 min trypsin treatment followed by twice washing the cells with cold PBS. Treatment with trypsin has been reported to effectively remove surface-bound and non-internalized CPPs [28]. We found that lysates prepared from trypsin -treated and cold PBS washed cells resulted in reduced ELISA signals compared to lysates prepared from cells that were washed with cold PBS alone (data not shown). We therefore adhered to former treatment throughout our study.

Although κFGF-MTS is considered relatively less effective than the cationic peptides [31], it has a unique advantage over cationic peptides; being hydrophobic in nature, κFGF-MTS does not rely on endocytosis for membrane transduction [32]. Studies of κFGF-MTS and other related hydrophobic signal sequence CPPs have shown that the secondary conformation is important for CPP transduction. A current model proposes that κFGF-MTS forms an α-helical hairpin, which interacts with lipids of plasma membrane. It subsequently unloops to a transmembrane conformation, penetrates the phospholipid bilayer, and transports the attached cargo into the cell [33]. This is advantageous since cargo attached to cationic peptides is known to get entrapped into endosomes with only a small percentage ending up in the cytosol [34]. We therefore used κFGF-MTS conjugated antibodies in all subsequent experiments. We employed live-cell confocal fluorescence microscopy to further confirm the internalization of Fluorescein -labelled κFGF-MTS-anti-p24 mAb into Jurkat and H9 T cells. The internalized antibody appeared to be present inside the cytoplasm and not associated with membranes.

Next we determined the highest concentration of κFGF-MTS-anti-p24 mAb that cells could be exposed to without experiencing cytotoxicity. We found that κFGF-MTS-anti-p24 mAb exhibited no detrimental effects on cell viability when used at 10 μg/1x10^5^ cells. Surprisingly, some native antibodies could also internalize into cells when co-incubated with cells at higher concentrations (20 and 40μg) and exhibited toxicity albeit lower than κFGF-MTS-anti-p24 mAbs. These results are in agreement with those reported by Zhao and co-workers who also reported some internalization of native antibodies into Jurkat T cells [24]. It could not be determined whether the native mAbs diffused passively into cells after a prolonged exposure or internalized actively via unknown receptors and mechanisms [24]. How mAbs (both native and κFGF-MTC -conjugated), when used in high concentration, exert toxicity towards cells warrants further investigation.

Cells pre-treated with κFGF-MTS-anti-p24 mAb showed markedly reduced virus replication when challenged with HIV-1. Exposure of cells to 10 μg/mL κFGF-MTS-anti-p24 mAb for 18 h prior to infection with HIV-1 resulted in ~50% inhibition of virus replication in both Jurkat and H9 T cells. Cells lysates subjected to pull-down assays demonstrated the interaction of κFGF-MTS-anti-p24 mAb with HIV Gag. These data suggest that internalized κFGF-MTS-anti-p24 mAb was responsible for HIV replication inhibition. When cells were continuously incubated with κFGF-MTS-anti-p24 mAb, inhibition of virus replication enhanced noticeably. This suggests that κFGF-MTS-anti-p24 mAbs continued to internalize into cells when maintained in culture medium at a constant concentrations. In control experiments, we treated cells with native anti-p24 and an unrelated κFGF-MTS-conjugated anti-AchE mAbs. No appreciable inhibition of virus replication observed in control experiments suggesting that virus inhibition was specifically due to cell treatment with κFGF-MTS-anti-p24 mAb, which internalized into the cells and apparently interacted with incoming viral capsid. To further confirm our results, we treated activated primary PBMCs with κFGF-MTS-anti-p24 mAb and infected antibody -treated cells with HIV-1. As anticipated, infectivity kinetics showed noticeably reduced HIV-1 replication in primary PBMCs that were treated with κFGF-MTS-anti-p24 mAbs prior to infection. Again, PBMCs treated with native anti-p24 and unrelated κFGF-MTS anti-AchE mAbs supported virus replication unabated.

In all these experiments we had treated cells with antibodies for 18 h prior to infection with HIV-1. This experimental setup shows the efficacy of antibody treatment towards the early events of virus replication. In the final experiment, we infected the PBMCs with HIV-1 and then treated them with antibodies. We did notice inhibition of virus replication in cells treated with κFGF-MTS-anti-p24 mAb but it was not as pronounced as it was in cells that were treated with antibodies prior to HIV infection. One explanation for this result is that the over-expression of Gag polyprotein by the infected cells overwhelmed the κFGF-MTS-anti-p24 mAb present inside those cells. Present data do not shed light on exactly how much κFGF-MTS-anti-p24 mAbs end up inside the cytoplasm and therefore warrants further investigation. However, the amount of antibodies ending up into cytoplasm might not be high enough to cope with the amount of Gag polyprotein synthesized.

We have identified a number of limitations in this study. First, the success rate of CPP-conjugation with antibodies could not be determined (we attempted to find out the percentage of CPP -conjugated antibodies using MALDI-TOF but the data was inconclusive), hence the correlation between κFGF-MTS-anti-p24 mAbs internalization level and HIV-1 inhibition were uncertain. Second, the chemical conjugation method is relatively inconsistent for it depends on multiple factors such as CPP purity, temperature, pH, incubation time, concentrations of chemicals, and availability of carbohydrate moieties on IgG molecule. At present we do not know which p24 epitopes this particular antibody is interacting with and whether those epitopes are present in the N- or C-terminal domains of p24. Both N- and C- terminal domains have been used to target p24 polymerization [35]. Based on the abovementioned issues, other alternatives such as generation of a cell internalizing antibodies by means of molecular linking of the gene sequence encoding the anti-p24 to κFGF-MTS (or other CPPs) and subsequent expression in suitable host (such as *E*. *coli* or mammalian cells) are being investigated by our group. We are also generating a panel of scFvs against p24 to map epitope binding regions and putative functional sites.

In this proof of concept study, we have shown that the κFGF-MTS-conjugated mAbs targeting HIV-1 p24 were able to efficiently inhibit the HIV-1 replication both in T cell lines and primary PBMCs. Inability of unconjugated anti-p24 mAb or an unrelated κFGF-MTS-conjugated anti-AchE mAb to inhibit viral replication showed that anti-viral activity was specifically due to κFGF-MTS-anti-p24 mAb. To the best of our knowledge, this is the second report demonstrating the viability of the intracellular HIV protein-targeting using antibodies. Zhuang and co-workers have recently engineered an anti-Rev Fab with Tat cell penetrating peptide and reported the antiviral activity of the cell internalizing FabRev1-Tat antibody against three different CCR5 isolates of HIV [36]. Since the antibodies described here majorly act on the early stage of HIV-1 cycle, it can be potentially adopted for HIV-1 prophylaxis or vaccination strategy.

## References

[1] Ensoli B, Cafaro A, Monini P, Marcotullio S, Ensoli F. Challenges in HIV Vaccine Research for Treatment and Prevention. Front Immunol. 2014; 5: 417 10.3389/fimmu.2014.00417 25250026 PMC4157563

[2] Emamzadeh-Fard S, Esmaeeli S, Arefi K, Moradbeigi M, Heidari B, Fard SE, et al Mechanisms of anti-retroviral drug resistance: implications for novel drug discovery and development. Infect Disord Drug Targets. 2013; 13(5): 330–336. 24712673 10.2174/1871526514666140321104049

[3] Willows S, Hou S, Hobman TC. RNA virus capsid proteins: more than just a shell. Future Virol. 2013; 8: 435–450.

[4] Ganser-Pornillos BK, Yeager M, Pornillos O. Assembly and architecture of HIV. Adv Exp Med Biol. 2012; 726: 441–465. 10.1007/978-1-4614-0980-9_20 22297526 PMC6743068

[5] Stremlau M, Owens CM, Perron MJ, Kiessling M, Autissier P, Sodroski J. The cytoplasmic body component TRIM5alpha restricts HIV-1 infection in Old World monkeys. Nature. 2004; 427(6977): 848–853. 14985764 10.1038/nature02343

[6] Ganser-Pornillos BK, Chandrasekaran V, Pornillos O, Sodroski JG, Sundquist WI, Yeager M. Hexagonal assembly of a restricting TRIM5alpha protein. Proc Natl Acad Sci USA. 2011; 108(2): 534–539. 10.1073/pnas.1013426108 21187419 PMC3021009

[7] Pertel T, Hausmann S, Morger D, Züger S, Guerra J, Lascano J, et al TRIM5 is an innate immune sensor for the retrovirus capsid lattice. Nature. 2011; 472(7343): 361–365. 10.1038/nature09976 21512573 PMC3081621

[8] Tang C, Loeliger E, Kinde I, Kyere S, Mayo K, Barklis E, et al Antiviral inhibition of the HIV-1 capsid protein. J Mol Biol. 2003; 327(5): 1013–1020. 12662926 10.1016/s0022-2836(03)00289-4

[9] Tian B, He M, Tang S, Hewlett I, Tan Z, Li J, et al Synthesis and antiviral activities of novel acylhydrazone derivatives targeting HIV-1 capsid protein. Bioorg Med Chem Lett. 2009; 19(8): 2162–2167. 10.1016/j.bmcl.2009.02.116 19297155

[10] Blair WS, Pickford C, Irving SL, Brown DG, Anderson M, Bazin R, et al HIV capsid is a tractable target for small molecule therapeutic intervention. PLoS Pathog. 2010; 6(12): e1001220 10.1371/journal.ppat.1001220 21170360 PMC3000358

[11] Jin Y, Tan Z, He M, Tian B, Tang S, Hewlett I, et al SAR and molecular mechanism study of novel acylhydrazone compounds targeting HIV-1 CA. Bioorg Med Chem. 2010; 18(6): 2135–2140. 10.1016/j.bmc.2010.02.003 20188575

[12] Shi J, Zhou J, Shah VB, Aiken C, Whitby K. Small-molecule inhibition of human immunodeficiency virus type 1 infection by virus capsid destabilization. J Virol. 2011; 85(1): 542–549. 10.1128/JVI.01406-10 20962083 PMC3014163

[13] Curreli F, Zhang H, Zhang X, Pyatkin I, Victor Z, Altieri A, et al Virtual screening based identification of novel small-molecule inhibitors targeted to the HIV-1 capsid. Bioorg Med Chem. 2011; 19(1): 77–90. 10.1016/j.bmc.2010.11.045 21168336 PMC3034313

[14] Lemke CT, Titolo S, von Schwedler U, Goudreau N, Mercier JF, Wardrop E, et al Distinct effects of two HIV-1 capsid assembly inhibitor families that bind the same site within the N-terminal domain of the viral CA protein. J Virol. 2012; 86(12): 6643–6655. 10.1128/JVI.00493-12 22496222 PMC3393593

[15] Kortagere S, Madani N, Mankowski MK, Schön A, Zentner I, Swaminathan G, et al Inhibiting early-stage events in HIV-1 replication by small-molecule targeting of the HIV-1 capsid. J Virol. 2012; 86(16): 8472–8481. 10.1128/JVI.05006-11 22647699 PMC3421734

[16] Goudreau N, Lemke CT, Faucher AM, Grand-Maître C, Goulet S, Lacoste JE, et al Novel inhibitor binding site discovery on HIV-1 capsid N-terminal domain by NMR and X-ray crystallography. ACS Chem Biol. 2013; 8(5): 1074–1082. 10.1021/cb400075f 23496828

[17] Kortagere S, Xu JP, Mankowski MK, Ptak RG, Cocklin S. Structure-activity relationships of a novel capsid targeted inhibitor of HIV-1 replication. J Chem Inf Model. 2014; 54(11): 3080–3090. 10.1021/ci500437r 25302989 PMC4245176

[18] Zhang H, Zhao Q, Bhattacharya S, Waheed AA, Tong X, Hong A, et al A cell-penetrating helical peptide as a potential HIV-1 inhibitor. J Mol Biol. 2008; 378(3): 565–580. 10.1016/j.jmb.2008.02.066 18374356 PMC2695608

[19] Zhang H, Curreli F, Zhang X, Bhattacharya S, Waheed AA, Cooper A, et al Antiviral activity of α-helical stapled peptides designed from the HIV-1 capsid dimerization domain. Retrovirology. 2011; 8: 28 10.1186/1742-4690-8-28 21539734 PMC3097154

[20] Zhang H, Curreli F, Waheed AA, Mercredi PY, Mehta M, Bhargava P, et al Dual-acting stapled peptides target both HIV-1 entry and assembly. Retrovirology. 2013; 10: 136 10.1186/1742-4690-10-136 24237936 PMC3842668

[21] Muller S, Zhao Y, Brown TL, Morgan AC, Kohler H. TransMabs: cell-penetrating antibodies, the next generation. Expert Opin Biol Ther. 2005; 5: 237–241. 15757385 10.1517/14712598.5.2.237

[22] Munyendo WL, Lv H, Benza-Ingoula H, Baraza LD, Zhou J. Cell penetrating peptides in the delivery of biopharmaceuticals. Biomolecules. 2012; 2(2): 187–202. 10.3390/biom2020187 24970133 PMC4030843

[23] Zhao Y, Lou D, Burkett J, Kohler H. Chemical engineering of cell penetrating antibodies. J Immunol Methods. 2001; 254: 137–145. 11406159 10.1016/s0022-1759(01)00410-0

[24] Zhao Y, Brown TL, Kohler H, Muller S. MTS-conjugated-antiactive caspase 3 antibodies inhibit actinomycin D-induced apoptosis. Apoptosis. 2003; 8(6): 631–637. 14739608 10.1023/A:1026139627930

[25] Loftus JC, Yang Z, Tran NL, Kloss J, Viso C, Berens ME, et al The Pyk2 FERM domain as a target to inhibit glioma migration. Mol Cancer Ther. 2009; 8(6): 1505–1514. 10.1158/1535-7163.MCT-08-1055 19509258 PMC3180876

[26] Wu F, Fan S, Martiniuk F, Pincus S, Müller S, Kohler H, et al Protective effects of anti-ricin A-chain antibodies delivered intracellularly against ricin-induced cytotoxicity. World J Biol Chem. 2010; 1(5): 188–195. 10.4331/wjbc.v1.i5.188 21541003 PMC3083952

[27] Teow SY, Mualif SA, Omar TC, Wei CY, Yusoff NM, Ali SA. Production and purification of polymerization-competent HIV-1 capsid protein p24 (CA) in NiCo21(DE3) *Escherichia coli*. BMC Biotechnol. 2013; 13: 107 10.1186/1472-6750-13-107 24304876 PMC4235032

[28] Richard JP, Melikov K, Vives E, Ramos C, Verbeure B, Gait MJ, et al Cell-penetrating peptides. A reevaluation of the mechanism of cellular uptake. J Biol Chem. 2003; 278(1): 585–590. 12411431 10.1074/jbc.M209548200

[29] Khan MA, Aberham C, Kao S, Akari H, Gorelick R, Bour S, et al Human immunodeficiency virus type 1 Vif protein is packaged into the nucleoprotein complex through an interaction with viral genomic RNA. J Virol. 2001; 75(16): 7252–7265. 11461998 10.1128/JVI.75.16.7252-7265.2001 PMC114961

[30] Vandegraaff N, Kumar R, Burrell CJ, Li P. Kinetics of human immunodeficiency virus type 1 (HIV) DNA integration in acutely infected cells as determined using a novel assay for detection of integrated HIV DNA. J Virol. 2001; 75(22): 11253–1160. 11602768 10.1128/JVI.75.22.11253-11260.2001 PMC114708

[31] Kabouridis PS. Biological applications of protein transduction technology. Trends Biotechnol. 2003; 21(11): 498–503. 14573363 10.1016/j.tibtech.2003.09.008 PMC2597147

[32] Orange JS, May MJ. Cell penetrating peptide inhibitors of Nuclear Factor-kappa B. Cell Mol Life Sci. 2008; 65(22): 3564–3591. 10.1007/s00018-008-8222-z 18668204 PMC2975941

[33] Veach RA, Liu D, Yao S, Chen Y, Liu XY, Downs S, et al Receptor/transporter-independent targeting of functional peptides across the plasma membrane. J Biol Chem. 2004; 279(12): 11425–114531. 14699109 10.1074/jbc.M311089200

[34] Erazo-Oliveras A, Muthukrishnan N, Baker R, Wang TY, Pellois JP. Improving the endosomal escape of cell-penetrating peptides and their cargos: strategies and challenges. Pharmaceuticals (Basel). 2012; 5(11): 1177–1209. 24223492 10.3390/ph5111177 PMC3816665

[35] Lemke CT, Titolo S, von Schwedler U, Goudreau N, Mercier JF, Wardrop E, et al Distinct effects of two HIV-1 capsid assembly inhibitor families that bind the same site within the N-terminal domain of the viral CA protein. J Virol. 2012; 86(12): 6643–6655. 10.1128/JVI.00493-12 22496222 PMC3393593

[36] Zhuang X, Stahl SJ, Watts NR, DiMattia MA, Steven AC, Wingfield PT. A cell-penetrating antibody fragment against HIV-1 Rev has high antiviral activity: characterization of the paratope. J Biol Chem. 2014; 289(29): 20222–20233. 10.1074/jbc.M114.581090 24878961 PMC4106338

